# Being an editor: then and now

**DOI:** 10.1128/aem.00066-26

**Published:** 2026-02-10

**Authors:** Kenneth Nickerson

**Affiliations:** 1 School of Biological Sciences, University of Nebraska 14719, Lincoln, Nebraska, USA; Michigan State University, East Lansing, Michigan, USA

**Keywords:** editor, reviewer

## Abstract

This commentary focuses on my experiences as an editor of *Applied and Environmental Microbiology* (AEM) from 1988 to 1996. I reflect on the challenges of the pre-internet world when all communications, including paper manuscripts, traveled by post. It was a time when editors chose the reviewers without the “benefit” of reviewers suggested by the authors. I describe the advantages and disadvantages of being an editor in those times, using seven memorable papers as examples of our efforts to advance both the field and our esteemed journal. I do so in the hope that this perspective brings relevance to present-day authors, reviewers, and editors.

## COMMENTARY

Hi folks. For most of my academic career, I have considered *Applied and Environmental Microbiology* (AEM) to be “my journal.” In the span of a 52-year career at the University of Nebraska-Lincoln, I have published 36 publications in AEM (1974–2024), served for 26 years on the journal’s editorial board of reviewers (1982–2008), and completed 9 years as an editor (1988–1996). My top three highly cited papers were published in AEM. The top two concerned our discovery of farnesol as the first eukaryotic quorum-sensing molecule (1, 2), whereas the third described a series of broad host range bacteriophages (3). However, it is my years as an editor for which I have been asked to tap my memory banks. In doing so, I hope to share a historical perspective of who we were and how we have grown to support our field, our journal, and the American Society for Microbiology (ASM).

In 1988, I joined six other AEM editors, all US based, all male, four with Robert as a first name, with Lars Ljungdahl as our editor in chief. John Bell, our production editor, distributed the incoming manuscripts evenly among the seven editors, each of us handling 200–220 manuscripts per year. In the pre-internet age, that meant a barrage of green and white envelopes with complete manuscripts going all over the world, to and from reviewers, authors, and ASM headquarters in Washington, DC. I remember the “thrill” of already feeling overworked only to discover five more incoming manuscripts in my mailbox. Remarkably, our turnaround time for responding to authors was usually only 3–4 weeks, which compares favorably with the 35-day average reported on AEM’s current webpage.

## THE OFFICE

At that time, ASM did not pay the editors. They did fund a part-time secretary/office assistant, a huge amount of postage, and a typewriter with sufficient memory to retain five form letters, one for each editorial decision: accept, minor modifications, major modifications, reject encouraging, and reject non-encouraging. It was always the middle paragraph that was important and distinctive for each manuscript. My estimate is that over those 9 years, we had 2% accept, 50% reject, and 48% modify and later accepted. I was fortunate to have a highly overqualified editorial assistant in my wife, Ann Weinkauff Nickerson (1942–2024), PhD from Wisconsin with Kenneth B. Raper. She helped greatly with many of the papers in the Mycology section of AEM for which we had primary responsibility. Also, I never had to worry whether names like *Phytophthora* or *Dictyostelium* were misspelled. Together, Ann and I scrounged for needed office items such as a postal scale to ensure that each green and white envelope leaving Lincoln had the correct postage. That scale is still in my garage today. ASM did not provide any formal instruction on how to be a good editor. I think they assumed that if you had published extensively, that meant you already knew what a good editor did. Thus, when I went back to my former post-doctoral home (the USDA lab in Peoria), seeking advice from Bob Hespell, we spent most of our time discussing how to set up an efficient office. Similarly, when John Leslie, who followed me as the AEM editor for Mycology, came to Lincoln in 1997, he spent much of his time with Ann learning office details.

## CHOOSING REVIEWERS

The most important job an editor had was choosing appropriate reviewers. I viewed the editor as the Judge, with the reviewers constituting 2–3 lawyers arguing for or against the acceptability of a manuscript. Reviewers were expected to be polite and helpful, even when pointing out deficiencies sufficiently serious to warrant rejection. Only very rarely did I have to rewrite or redact an inappropriate review. Remember that these selections had to be made in an era before the authors provided a list of suggested reviewers. However, we editors had the huge advantage of a highly qualified editorial board (then 134 members, now over 200), as described in 37 yellow pages detailing their expertise and areas they felt comfortable reviewing. The keyword here is “detailing.” Board members described their expertise, specifying organisms, metabolic pathways, enzymes, ecosystems, experimental approaches, and overall research goals. In short, they accurately depicted why they had been selected for the editorial board. Board members could expect to review 30–40 papers. Over the years, strong editor-reviewer bonds would develop, oftentimes without ever having met in person. For instance, Bryan White (Illinois) was my favorite reviewer for cellulose degradation, Ken Hammel (Wisconsin) for lignin degradation, Mike Adang (Georgia) for microbial insecticides, Shelby Freer (USDA) for fungal physiology, and Allen Phillips (Penn State) for enzyme kinetics.

The expertise of the editorial board, even as broad as it was, often did not fit the variety of the submissions. The *raison d’être* of AEM was to cover all areas of microbiology. It provided a home for everything not covered by the other more narrowly focused ASM journals. The AEM editors relished this diversity, which was especially evident every August when editors not on vacation covered manuscripts in areas usually assigned to editors who were on vacation. The net result was that 1/3 of the reviewers I chose were not from the editorial board. I still have an alphabetized shoe box containing roughly 500 cards describing these *ad hoc* reviewers. My favorite strategy was to examine the reference section of the manuscript in question, looking for a recent relevant review, or the first or corresponding author of a similar but non-overlapping paper. Remarkably, in this pre-internet age, I received thoughtful reviews from ca. 70% of these *ad hoc* requests! I attribute this high success rate to the collective esprit de corps of the microbial research community at that time and their awareness of the upward trajectory in career paths: published author to *ad hoc* reviewer to editorial board member to editor to editor in chief. It probably also helped that at that time, submissions were generally smaller, aiming for five printed pages, without extensive supplementary material.

## ENGAGING REVIEWERS

A good reviewer is worth their weight in gold. Some strategies I used included the following. (i) Do not overuse them. You do not know how many manuscripts they receive from other editors. (ii) Make it clear they should only evaluate the science. They do not need to correct or upgrade English usage. At that time, ASM employed some highly competent copy editors prior to publication. (iii) If the English was just too bad, they should simply return it unreviewed. (iv) Do not force them to look at every set of changes made in modified papers. It is a waste of their valuable time. A competent editor can evaluate whether the authors have done what they were supposed to do. (v) Whenever possible, follow a reviewer’s strong recommendation. My belief is that editors, especially AEM editors, had to be generalists, whereas the reviewers, if properly chosen, were the real experts in their sub-disciplines. One of my friends, an eventual National Academy member, quit the AEM editorial board because a different editor twice overruled their recommendation to reject. (vi) In an emergency, step in as reviewer #2 or 3 yourself. (vii) When a paper has multiple different topic areas, for example, bacteriophages infecting sulfate-reducing bacteria (SRBs) as they influence the sands surrounding oil fields, tell the reviewer chosen for expertise in bacteriophages that the other areas will be covered by the other reviewers. (viii) Expect surprises. For instance, I once chose a reviewer because of the quality of a paper listing them as the first author. The reviewer later mentioned that they had been surprised by the request because at the time they were only a second-year graduate student. Or the reviewer who apologized for the delay in returning their review because their spouse had just died. It then transpired that they were both over age 90, but the reviewer, who had been President of an industrial baker’s yeast association, had just the right expertise I needed for that paper. And my favorite, a series of handwritten reviews from John Pitt, a famed mycologist and expert in food spoilage. All his reviews were written during flights from Australia to Singapore and mailed from Singapore. They were all quality reviews.

## SEVEN MEMORABLE PAPERS

In thinking about memorable papers I have handled, I have chosen seven that illustrate the microbial diversity inherent in AEM and the flexibility required of an editor.

1. Welcome to AEM (4). These researchers collected 2,886 healthy bats from 12 widely scattered sites in the Amazon Basin of Brazil. They removed the livers, spleens, and lungs and then determined the fungal presence in each of the organs. A total of 5.4% of the bats were fungal carriers. A total of 71 bat species contained 38 fungal species, including 10 *Candida* spp. The authors concluded that neotropical bats in the Amazonian ecosystem serve as fungal carriers and/or natural habitats for yeasts and yeast-like fungi. Monumental.

2. Six years after Chernobyl (5). The authors examined 41 collections of edible mushrooms (25 from the Ukraine, 6 from Sweden, 8 from Ontario, and 2 from Michigan) for their ^134^Cs/^137^Cs levels. Radioactivity in the Ukrainian and Swedish mushrooms averaged 23 and 48 times more than that of the North American mushrooms. Radioactivity was especially high in the mycorrhizal species. Eat mushrooms at your own risk.

3. An average human life span of less than 40 years (6)? Aflatoxins are just about the most potent mutagens, carcinogens, and teratogens known. The Paharia tribe lives in a moist, hilly region of Bihar State in India, where they grow and store pearl millet as a major part of their diet. Following the rainy season of 1989, the dominant mold flora of stored millet was *Aspergillus flavus* and *Aspergillus parasiticus*, while the levels of aflatoxin B_1_ in stored and cooked millet ranged from 23 to 2,110 and from 21 to 549 ppb, respectively. This was a story that needed to be told.

For these first three publications, the data presented were the memorable feature. In retrospect, their impact on human health may have contributed to my mid-career shift from *Bacillus thuringiensis* and microbial insecticides to *Candida albicans* and fungal quorum-sensing molecules. The next four examples illustrate areas of editorial discretion.

4. Paul Baumann’s minireview on mutualistic associations of aphids and prokaryotes (7). Minireviews were a comparatively new feature, attractive for journal competitiveness. Each editor was supposed to solicit and handle them; a dedicated editor for minireviews was far in the future. In 1994, AEM approved Invertebrate Microbiology as a new section. AEM had published hundreds of papers on *B. thuringiensis* and other microbial insecticides, and I had been chosen as an editor in large part because of my expertise in this area. The section name Invertebrate Microbiology was a compromise. Clearly, invertebrates were not microbial and vice versa. Insect Microbiology was deemed too narrow, and All Interactions Among Microbes, Insects, and Other Invertebrates was too cumbersome. Since I had led the fight for this new section, I wanted it to get off to a good start. A minireview by one of my favorite authors and reviewers was indicated. Fortunately, Paul agreed, and the review (7) inaugurating the new section appeared as pages 1–7 for AEM in 1995.

5. Page limits and a chemostat (8). I had in hand a minireview from a group of Dutch microbiologists led by Hans van Dijken. It concerned the use of continuous culture to study transport systems in yeasts. The reviewers loved it, but they also suggested some clarifications and improvements. They also pointed out that it was much too long to be a minireview in AEM. In the age of print journals, we had to take page limits seriously. When the revised manuscript came back, it was great scientifically, but now it was two pages longer. What to do? After some thought, I called the editor in chief of *Microbiological Reviews*, whom I barely knew, and explained my dilemma. He agreed to a transfer from AEM to MR (now known as *Microbiology and Molecular Biology Reviews* or MMBR). With the enthusiastic approval of the authors, the review article (15 pages) was published in MR without further review a few months later.

6. An appropriate way to impress an editor?? Not every paper is a clear-cut yes or no. For another paper with a different group of Dutch authors, I had followed the consensus of three reviewers and sent them a reject-encouraging decision letter. Then, a few days later, I received a message from one of the authors saying that after extensive discussions (in a bar until late at night), they had decided that the reviewers I had chosen were incompetent, and I was an IDIOT (their word and capitalized font format). Clearly, this is not a standard method for gaining favor with an editor. However, while I disagreed with their overall assessment of my abilities, I was impressed with their arguments and retracted my early decision. Their paper was later published after modest revisions.

7. An untimely death (9). Single-authored papers were already unusual by 1991. My difficulty concerned a submission by the Australian Alan Warth on the antifungal effects of benzoic acid on yeasts, as assessed via measuring pH_i_ and glycolytic carbon flow. The reviewers liked the paper but requested significant, albeit non-major, changes (i.e., no further experiments were required). As usual, we requested the revisions within 2 months, and when they did not arrive on time, I made inquiries and then learned that Alan had just died. What to do? He had no co-authors and no close colleagues to assist. I finally decided to write the needed revisions myself. His departmental chair was very cooperative. A key feature was whether the glossy figures could be found. At that time, figures were not computer generated. Most research institutions had a full-time artist on staff to convert hand-drawn figures into professional “glossies” suitable for publication, but these were often only prepared and submitted just prior to publication. Thus, did they exist, and could they be found? Fortunately, they were located and coupled with my revisions, and the paper was published (9). Alan’s chair suggested that I should be a co-author on the paper, which I respectfully declined, choosing instead to be named in the acknowledgments section. As compensation, they sent me a very nice copy of Foodborne Microorganisms of Public Health Significance (10). Years later, some references still list this paper as by Warth and Nickerson.

## ADVANTAGES AND DISADVANTAGES

Let’s do the easy one first. For disadvantages, there is only one, but it is a biggie: time constraints. Over 200 manuscripts per year means at least four new assignments every week, and perhaps more in August to cover for editors on vacation. Thus, editorial duties took up 40% of my time, while maintaining my normal teaching load of one course per semester at my institution. In contrast, the advantages were far more diverse. My son Daniel, also a yeast biologist, has described this commentary as a love letter to AEM. Being an editor serves to validate that one has been reasonably successful in one’s chosen field. It enhances your chances for promotion, collaborations, consultantships, and getting your own research funded and published. You can help craft the research subdivisions that you cover for AEM. You can improve the visibility of microbiology at your university by well-chosen recommendations for the editorial board. Notably, recognition as an AEM editor enhances your own visibility in the field and expands your career opportunities if you are so inclined.

But I have saved my favorite advantage for last. Being an editor broadens your expertise as a research scientist and teacher. In my years at the University of Nebraska-Lincoln, I have taught graduate courses in Microbial Diversity, Microbial Physiology, and Industrial Microbiology, as well as the more medical introductory courses. Quickly, each of these courses benefitted from real-life examples, which I gleaned from recent AEM papers, especially for the Diversity course. As for research, I was a chemist who became a biochemist who became a microbiologist, but only in my term as an editor did I feel that I was a microbiologist. A research case in point concerns SRBs. One time, probably in August, I got a paper to handle on SRBs. As a matter of self-preservation, and to do justice to the authors, I got a copy of John Postgate’s excellent little book on SRBs (11) and read it carefully. This precaution helped in a favorable review and had huge ramifications when I later consulted for a major manufacturer of center pivot irrigation systems. In some locations, their stainless steel center pivots, fully warranted to perform for 10 years, were collapsing of their own weight after only a year. Who knew that stainless steel could actually be cut by a butter knife? But, having just read Postgate’s book, I was able to diagnose anaerobic metal corrosion at neutral pH by SRBs, which could be easily prevented by lining the pipe interior with a polymer to protect the stainless steel. I charged them $5K for my services, and later was told that my recognizing the role of SRBs in corrosion had saved them $50 million and allowed them to avoid bankruptcy.

## BROADER HORIZONS (aka DIVERSITY)

One of the most important and pleasant tasks an editor had at the end of each year was to recommend individuals for the editorial board. Typically, I recommended 10–12 who had impressed me either as an author or reviewer. These choices were investments in the future. When I left in 1997, Judy Wall was the editor in chief, and the four Roberts had been replaced by Gary Roberts and eight other new editors (total = 10). The editorial board now had 160 members, and I had recommended roughly 20%–25% of them. Several of my recommendations later became editors, and one became editor in chief. Now, with Gemma Reguera as editor in chief, AEM has 24 Editors (13 female) representing 12 countries, with a dedicated minireview editor. Furthermore, the journal is supported by a dedicated editorial board of over 200 reviewing members, who are selected from existing board or *ad hoc* reviewers using an inclusive model of membership based on annual review contributions (at least three per year) (12).

In addition to broader geographic horizons, it is nice to note the inclusion of researchers with non-university affiliations. The USDA had long been well represented, but now we have editors from the industry, as well as from the EPA, CDC, Oak Ridge National Laboratory, and agencies directed toward food safety, forest products, and fisheries. I also have fond memories of Jim Spain (US Air Force) as an editor. Think about the biodegradability of the compounds used for deicing airplanes. Individuals in military uniforms are rarely seen at ASM meetings, but I note that several of the students I have mentored with my long-term colleague Audrey Atkin have chosen careers as microbiologists in the military, while one of our MS students was an active duty army officer during his 2 years at UNL. This overlap seems natural to me because my father (Walter J. Nickerson) spent 4 years in World War II (1942–1946) researching fungal infections of the feet and other microbial aspects of jungle warfare. These studies led to sandals, Desenex, and methods for tropic proofing cloth. He said that this experience was equivalent to an excellent 4-year post-doc at a time when actual post-docs were scarce. It also provided the material for his book, Biology of Pathogenic Fungi (13), which set the tone for the rest of his career. All that broad expertise is recognized and represented at AEM. The editorial process may have changed throughout the years, but AEM remains the home for those of us building a field and community that crosses scientific boundaries to inspire the next generation of scientists in AEM.

## References

[1] Hornby JM, Jensen EC, Lisec AD, Tasto JJ, Jahnke B, Shoemaker R, Dussault P, Nickerson KW. 2001. Quorum sensing in the dimorphic fungus Candida albicans is mediated by farnesol. Appl Environ Microbiol 67:2982–2992. doi:10.1128/AEM.67.7.2982-2992.2001 11425711 PMC92970

[2] Nickerson KW, Atkin AL, Hornby JM. 2006. Quorum sensing in dimorphic fungi: farnesol and beyond. Appl Environ Microbiol 72:3805–3813. doi:10.1128/AEM.02765-05 16751484 PMC1489610

[3] Jensen EC, Schrader HS, Rieland B, Thompson TL, Lee KW, Nickerson KW, Kokjohn TA. 1998. Prevalence of broad-host-range lytic bacteriophages of Sphaerotilus natans, Escherichia coli, and Pseudomonas aeruginosa. Appl Environ Microbiol 64:575–580. doi:10.1128/AEM.64.2.575-580.1998 9464396 PMC106085

[4] Mok WY, Luizão RC, Barreto da Silva M do S. 1982. Isolation of fungi from bats of the Amazon basin. Appl Environ Microbiol 44:570–575. doi:10.1128/aem.44.3.570-575.1982 6890326 PMC242059

[5] Smith ML, Taylor HW, Sharma HD. 1993. Comparison of the post-Chernobyl 137Cs contamination of mushrooms from eastern Europe, Sweden, and North America. Appl Environ Microbiol 59:134–139. doi:10.1128/aem.59.1.134-139.1993 8439144 PMC202067

[6] Mishra NK, Daradhiyar SK. 1991. Mold flora and aflatoxin contamination of stored and cooked samples of pearl millet in the Paharia tribal belt of Santhal paragana, Bihar, India. Appl Environ Microbiol 57:1223–1226. doi:10.1128/aem.57.4.1223-1226.1991 1905519 PMC182872

[7] Baumann P, Lai C-Y, Baumann L, Rouhbakhsh D, Moran NA, Clark MA. 1995. Mutualistic associations of aphids and prokaryotes: biology of the genus Buchnera. Appl Environ Microbiol 61:1–7. doi:10.1128/aem.61.1.1-7.1995 16534896 PMC1388313

[8] Weusthuis RA, Pronk JT, van den Broek PJ, van Dijken JP. 1994. Chemostat cultivation as a tool for studies on sugar transport in yeasts. Microbiol Rev 58:616–630. doi:10.1128/mr.58.4.616-630.1994 7854249 PMC372984

[9] Warth AD, Nickerson KW. 1991. Mechanism of action of benzoic acid on Zygosaccharomyces bailii: effects on glycolytic metabolite levels, energy production, and intracellular pH. Appl Environ Microbiol 57:3410–3414. doi:10.1128/aem.57.12.3410-3414.1991 1785916 PMC183988

[10] Buckle KA. 1989. Foodborne microorganisms of public health significance. 4^th^ Edition. Australian Institute of Food Science and Technology Ltd.

[11] Postgate JR. 1979. The sulphate-reducing bacteria. Cambridge University Press.

[12] Reguera G. 2023. A new model for inclusive membership in the AEM editorial board. Appl Environ Microbiol 89:e01402-23. doi:10.1128/aem.01402-23

[13] Nickerson WJ. 1947. Biology of pathogenic fungi. The Ronald Press Company, New York.

